# Assessing the impact of case mobility: issues and recommendations from Greece

**DOI:** 10.1186/s44147-021-00003-1

**Published:** 2021-07-26

**Authors:** Athanasios Thanos Giannopoulos

**Affiliations:** TREDIT (Transeuropean Consultants for Transport, Development, and Information Technology) S.A., 78C Vryoulon & K. Karamanli St, 55132 Thessaloniki, Greece

**Keywords:** ITS evaluation, CASE mobility, Transport assessment, Autonomous transport, Evaluation, Mobility as a Service

## Abstract

This paper is concerned with the assessment of future applications of CASE (Co-operative, Autonomous, Shared, and Electric) mobility—a term that is also taken to include the more traditionally known applications of ITS (Intelligent Transport Systems). It sets the objective of making such assessments more holistic and horizontal in nature because future CASE mobility applications will include many technologies and service concepts as an integrated whole serving specific mobility objective. Traditional evaluation methodologies will therefore have to be modified to account for this situation, and to this end, the paper focuses on assessing and adapting such “traditional” methodologies. It draws from the experience gained in Greece in the last decade when a substantial number of ITS applications were implemented and assessed, especially in the second largest urban area of the country, the city of Thessaloniki (part of the EU’s European Network of Living Labs). Four basic methodologies are selected: the use of KPIs (Key Performance Indicators), focused interviews, the CMME (CASE Mobility Matrix Evaluation), and the use of safety audits before and after the CASE mobility application. For the first three, the paper suggests specific indicators and/or content. It also gives an example of the use of CMME based on a use case from Thessaloniki. The contents and recommendations of this paper provide a better understanding of the emerging situation as regards CASE mobility applications and point to the need for establishing a timely and comprehensive CASE mobility evaluation framework at both national and European levels, for future implementations.

## Introduction

### Definitions and objectives

The acronym “CASE” stands for Co-operative, Autonomous, Shared, and Electric but the term “CASE mobility” that is used in this paper denotes not only these four advanced forms of urban mobility but also all Information Technology (IT) or Artificial Intelligence (AI)-based applications that form the so-called Intelligent Transport Systems (ITS), or the connected intelligent transport systems (C-ITS) as well as other non-transport, but related, technologies such as the Internet of Things (IoT) or the big data collection and analysis infrastructures [1, 2]. Urban mobility issues will become again (after the COVID-19 pandemic) one of the main priorities especially since more than 75% of the population of Europe live in urban areas and this is expected to increase to almost 84% by 2050 (see the page “Urbanisation in Europe” in https://knowledge4policy.ec.europa.eu/). CASE mobility became, for the first time, the subject of an official EU policy document in the Urban Mobility Package (UMP) of 2013 where the coordinated deployment of urban ITS, became one of the five key focus policy areas (see https://ec.europa.eu/transport/themes/urban/urban-mobility/urban-mobility-package_en). In the same package, there were provisions for the development and implementation of Sustainable Urban Mobility Plans (SUMPs) for each urban area of the EU with over 100 000 population. These plans include measures and policies for the development of sustainable mobility, several of which fall under the CASE mobility category [3]. Already hundreds of European urban areas have developed such SUMPs.

Evaluation and assessment methodologies for the sustainable urban mobility applications have been discussed in connection to measures in the Urban Mobility Package [2] and the SUMPs [3]. They involve, (a) regular “quick scans” that provide structured guidance to city governments for improving their SUMPs and (b) specific evaluations for the assessment of economic, social, and environmental impacts [[2] , pp 165–200]. For the “quick scans,” the SUMP Self-Assessment tool has been introduced in February 2020 by the European Platform on Sustainable Urban Mobility Plans (see https://www.sump-assessment.eu—accessed November 2020). This tool is primarily qualitative and consists of eight sections with 30 to 45 questions depending on the specific planning context. SUMP assessment by the use of these methodologies became mandatory in the legislation of a number of EU-member countries [4]. Further to these, rather focused on SUMPs evaluation tools and methodologies, there have been a number of variations of evaluation methodologies for the, more conventional, ITS applications, and most of these can be found in [5].

More specialized evaluation methodologies have been proposed for the technical assessment and verification of the more advanced CASE mobility applications such as autonomous vehicles transport. In Europe, these methodologies were set by the German government-supported project PEGASUS (Project for the establishment of generally accepted quality criteria, tools, and methods as well as scenarios and situations for the release of highly automated driving functions—see: https://www.pegasusprojekt.de/en/ - accessed December 2020). These are standardized procedures for testing and experimenting with autonomous vehicles leading to the securing and approval of higher levels of automation. For the evaluation of other advanced types of CASE mobility applications such as shared transport or autonomous electric or last-mile autonomous transport services providing door-to-door services to public transport (PT) passengers, the European research project SHOW [6] is using two kinds of evaluation methodologies. The first involves economic and business impact assessments such as Go-to-Market viability or Total-Cost-of-Ownership analyses, Cost Benefit, and Cost Effectiveness assessments. The second type of evaluation methodologies used in project SHOW are more holistic impact assessment studies that will be performed in the more than 70 use cases of CASE mobility demonstrated in the project. These holistic assessments include (a) interviews using the Motivational Interviewing Competency Assessment (MICA) framework (http://micacoding.com/—accessed December 2020) and (b) Key Performance Indicators (KPIs) in the following impact areas: safety, traffic efficiency, energy consumption, environment, society (employability and equity), logistics operations, user experience, awareness building, and user acceptance.

The purpose of this paper is to propose evaluation methodologies for CASE mobility that are easy to implement and find data for, while at the same time are holistic enough to assess the impacts of CASE mobility applications that include several individual measures and services. In doing so, we utilize the experience gained by several CASE mobility applications in Greece to which we turn our attention in the next section.

### Case mobility applications in Greece

Thessaloniki (population 1 million) is the second largest city of Greece and the first to be designated as an EU “Living Lab” forming part of the European Network of Living Labs (ENoLL) [7]. As such, the city has—and still is—implementing smart mobility applications primarily through the Thessaloniki Smart Mobility Living Lab (TSMLL) of the Hellenic Institute of Transport (see https://smartmlab.imet.gr/ - accessed December 2020). The first of such applications were three C-ITS schemes implemented in 2012–2013 for energy-efficient intersection control, road hazard warnings, and violation of the red light monitoring [8]. The initial applications were later expanded with more CASE-related technologies which were all integrated in the so-called Urban Mobility Management System of Thessaloniki and were monitored via the central Traffic Control Centre installed and operated in the regional government offices (the Region of Central Macedonia) [9].

Data collection is performed in Thessaloniki on a regular basis as part of the Thessaloniki Living Lab infrastructure of sensors and counters which include Bluetooth detectors, ITS cooperative roadside units, on-board units on cooperative vehicles for collecting floating car data (taxis), inductive loops and cameras, smart traffic lights, and various social media-related application programs that relay information on trip making and other travel characteristics [10]. Social media is an increasingly applied method for trip data collection worldwide [11]. All these data form the “big data” for the mobility repository of the “Urban Mobility Management System of Thessaloniki” that is deposited in the Thessaloniki Smart Mobility Living Lab (TSMLL). The processing of these data enables the estimation, on a regular basis, of traffic flows, travel times, short-term traffic condition predictions, spatial expansion of traffic flows (where there are no counts), and real-time traffic characteristics estimation [12].

CASE mobility developments took place also in other Greek urban areas, most notably in the city of Trikala in central Greece. This is a city of approximately 60,000 population where “Smart Trikala” is the name given to the several CASE mobility applications implemented there, which include electronic GIS displays, online dynamic traffic light operation, autonomous bus operation (along one public transport line), a “smart” and connected digital platform that provides one central access point to all CASE applications in the city, operation of a new traffic control center for all the “Smart Trikala” city services, and others (https://trikalacity.gr/en/smart-trikala/—accessed December 2020).

The above two urban areas are expected to form in the not too distant future the first “smart cities” in Greece. Smart city development is a worldwide trend that has already started [13, 14].

## Methods

### Background

The experience gained from the CASE mobility applications in Greece (as briefly described above) is valuable not only in terms of the advanced technological performance of transportation networks and services that they provide, but also in terms of the appraisal of the applicability and effectiveness of the various evaluation methodologies used for their evaluation and assessment. As already indicated in the Introduction, historically speaking, there are many evaluation methodologies that have been used on various related occasions as for example with the ITS measures applied or the sustainable mobility measures and so on. We hold the view that CASE mobility applications, in the frame of a country like Greece where relevant data is scarce (in fact relevant adequate data exist only for the city Thessaloniki), would necessitate evaluation methodologies that abide with the following three principles: they are simple to use, require a minimum of data, and are reliable enough to allow adequately documented decision-making. Having reviewed several evaluation methodologies that were used in a number of CASE mobility applications that took place primarily in Thessaloniki, Greece, in the last decade (2010–2019), four basic CASE mobility evaluation methodologies are being proposed. These methodologies have been tested, in one form or another, in a number of CASE mobility applications that were part of EU co-funded research projects implemented in the city of Thessaloniki, Greece, by the authors’ company (TREDIT SA) or the Hellenic Institute of Transport (HIT/CERTH), both of which are based in Thessaloniki. Indicatively, these projects (listed in descending chronological order from the most recent ones, down) are the following: AVENUE (Autonomous Vehicles to Evolve to a New Urban Experience—https://h2020-avenue.eu/), C-MOBILE (Accelerating C-ITS Mobility Innovation and deployment in Europe—https://c-mobile-project.eu/), AEOLIX (Architecture for European Logistics Information exchange—https://aeolix.eu/), GALILEO4MOBILITY (Fostering the adoption of GALILEO for Mobility as a Service—http://www.galileo4mobility.eu/), IRIS (Integrated and Replicable Solutions for Co-Creation in Sustainable Cities—https://www.irissmartcities.eu/), TransAID (Transition Areas for Infrastructure-Assisted Driving—https://www.transaid.eu/), CAPITAL (Collaborative capacity Programme on ITS Training-education and Liaison—https://capital-project.its-elearning.eu/), CO-GISTICS (Cooperative logistics for sustainable mobility of goods—https://cogistics.eu/), COMPASS4D (Cooperative Mobility Pilot on Safety and Sustainability Services for Deployment—https://trimis.ec.europa.eu/project/ compass4d), and finally project CONDUITS (Coordination of network descriptors for Urban Intelligent Transportation Systems—one of the first projects to formulate and propose KPIs for ITS—https://cordis.europa.eu/project/id/218636). All sites are accessed December 2020.

### Possible methodologies for evaluating case mobility

#### The use of KPIs

This is a well-known method of assessing the performance and impacts of a specific technological or service application [5, 15–17]. The method has been used in Greece for ITS or C-ITS applications with good results so far [9, 15, 18]. The method consists of setting up a number of appropriately calculated indicators and then collecting data and calculating their values before and after implementation of the change that is being evaluated. For CASE mobility applications, the selection of the KPIs to use may be tricky due to the complex use cases that will often need to be evaluated (e.g., introduction of Mobility as a Service operations in an area). As a general rule, the KPIs to be used should:

- Describe sufficiently the two situations before and after the implementation of the CASE mobility application;
- Are commonly understood (and in the same way) by all stakeholders involved; and
- Are able to be quantified with existing, or easy to be collected, data.

The use of KPIs is strongly related to the existence of a reliable and permanent system of data collection and monitoring. This is normally the work of special mobility monitoring observatories which, for each major urban area, should be established in order to regularly and consistently map and collect CASE mobility-related data. The architecture for these data repositories should conform to Open Services and Open Data policies standards (so that they are accessible and available to all interested and authorized mobility service providers or mobility information provision agencies).

### Conducting a number of focused interviews

Such interviews are normally conducted with travelers, passengers of private or public (mass) transport vehicles, or drivers of private or professional vehicles, which are the second CASE mobility assessment methodology suggested. Securing the selection of a random set of persons to be interviewed (in numbers that are sufficiently representative of the total “population” in each category) is important. According to the experience gained by the Thessaloniki applications of the projects mentioned in the “Key Performance Indicators” section above and taking into account the recommendations of Topic study 5 of the EU funded project CAPITAL [19], three categories of interviewees are recommended (at minimum): taxi drivers, public transport (PT) riders, and private car drivers. The defining characteristics of these interviews are as follows:

1. Taxi drivers*.* These interviews must be based on a small set of key questions so as to incite the maximum response rate and minimize the time required for the answers. Indicative subjects of interview questions (referring to changes between the “before” and the “after” situation):

- Travel times (or congestion levels) along specific corridors;
- Estimated level of congestion along specific corridors and times of day;
- Changes in taxi energy consumption per time period (day or week); and
- Changes in (taxi) ridership levels.
  2. Bus or other mass public transport (PT) riders. These interviews aim at assessing changes in the quality of service, as perceived by the PT passengers, before and after the CASE mobility applications. Indicative subjects of interview questions (referring to changes between the “before” and the “after” situation):
- Overall quality of service of the PT system used (distinguish by line or corridor);
- In-vehicle travel times (distinguish by line or corridor);
- Waiting times at stops (distinguish by line or corridor); and
- Quality of door to door overall travel.
  3. Private car drivers or passengers*.* Indicative subjects of interview questions (referring to changes between the “before” and the “after” situation):
- Travel times (distinguished by corridor);
- Ease of parking at destination;
- Walking times to final destination;
- Ease of routing and navigation; and
- Information provision.

#### Road Safety Audits before and after

For a more “road safety focused” evaluation, and in addition to the more “traditional” before and after KPI comparisons suggested earlier, a more in-depth evaluation of the safety implications of CASE mobility to a whole road axis or network segment, may be achieved through a road safety audit (see https://safety.fhwa.dot.gov/rsa/—accessed December 2020) before and after the application(s). Road safety audits can give a good measure of the impact of CASE mobility in an area-wide sense by providing results that assess:

- The number of elements of the road infrastructure that represent a safety concern before and after the CASE application;
- To what extend the implemented CASE mobility application(s) have eliminated or mitigated previously identified safety concerns;
- Other changes in the (road) safety levels e.g., which changes for which road users, or impacts on vulnerable road users (VRUs), etc.

#### Matrix Evaluations (qualitative)

For cases where there are not sufficient data available or there are elements that cannot be expressed through KPIs, a more horizontal and qualitative approach is recommended. This is the CASE Mobility Matrix Evaluation or CMME approach. It involves the construction of relational matrices showing the qualitative assessment in terms of the potential impacts foreseen for each of a number of separate elements that constitute the specific CASE application. The evaluation table has as rows the various categories of expected impacts and as columns the elements that constitute the CASE application(s) being evaluated. When using the CMME approach in order to compare two alternative CASE mobility applications, elements referring to both applications are included in Table 1 (see example below). Each of the rows of the CMME table represents potential impacts. These can also be thought off as the criteria for the assessment. For each such criterion (potential impact) and element of the CASE application, a score value (normally from 1, i.e., minimum impact, to 10, i.e., maximum impact) is assigned by a group of “evaluators” which consists of a small number of experts familiar with the area and new CASE mobility applications. Also, each criterion is assigned a “weight” (normally a number from 1 to 3 or more) expressing its importance and gravity. The process is similar to that of multi-criteria analysis (MCA). The total sum of the products, scores by weights, gives a measure of the “evaluation impact” and by comparing the total scores of alternative CASE applications an ex-ante evaluation of their impact and importance can be achieved. Of critical importance in the whole process is the split that will be made of the overall CASE application into its various elements (columns of the CMME table). A representative way to do that can be derived from the analyses made in the EU-funded research project CAPITAL [19]. Also, important are the various impact categories that will be included in the rows of the CMME table.

**Table 1:** KPIs suggested for CASE mobility applications

A/A	Groupings (Impact on:)	Name of indicator	Description
1	Public transport operations (by mode of PT, e.g., buses, trams, metro)	Total no. of PT passengers (by mode)	Total number per select time period, before and after CASE application
2		PT occupancies	Average no. of passengers per PT vehicle before and after
3		PT reliability	% of schedule reliability (in a select no. stops
4		Average PT speed	Average speed over select no. of routes
5	Air pollution—environment	CO_2_ emissions from car traffic	Measurements of CO_2_ concentrations at select no. of points by the road, before and after the CASE applications
6		NOx emissions (per capita)	Measurements of NOx concentrations at select no. of points by the road, before and after the CASE applications
7		Total hydrocarbon energy consumption of private vehicles per day	The total consumption in terms of tons of hydrocarbon fuel estimated by the total number of vehicles using such fuel in the network for a typical 24-h period multiplied by the average fuel consumption
8		PM_10_ and PM_2.5_ emissions (per capita)	Measurements of PM_10_ and PM_2.5_ concentrations at select no. of points by the road, before and after the CASE applications
9	Accessibility—mobility	Average time to reach a PT stop	Average times to reach (by walking or other means) a select number of PT stops before and after
10		Average travel times over a select no. of routes	% change in average travel times over a select no. of routes before and after
11		Average waiting times at bus stops	Average waiting times at a select number of bus stops before and after the CASE applications
12		% usage of non-motorized transport modes	% of daily trips using bicycles or walking before and after the CASE applications
13		Occupancy rates of private vehicles	Occupancy rates of private vehicles before and after the CASE applications
14	Road safety	No of road accidents	No of total road accidents before and after the CASE applications
15		No. of fatalities in road accidents	No. of fatalities in road accidents before and after the CASE applications
16		No. of accidents involving vulnerable road users	No. of accidents involving vulnerable road users before and after the CASE applications
17		Safety of the Intended Functionality (SOTIF) (https://semiengineering.com/expanding-automotive-safety-with-sotif/—accessed December 2020).	For safety assessments in the case of autonomous transport applications in order to assess the risk resulting from functional failures or inefficiencies of the intended functionality

The subjectivity that pervades the CMME process can be a matter of concern. This subjectivity stems from the (inevitably subjective) choices that have to be made by the experts who fill the relevant scores in the CMME tables, and their weights. While this is true, the method can nevertheless bring a degree of structured analysis and openness to the evaluation of CASE mobility, analogous to the one achieved in decision-making by the use of MCA. In any case, these “subjectivity concerns” can be substantially eased by the fact that the choices are not made by one person but by a group of experts working in coordination and following a consensus formation process that has been pre-established.

## Results

### Key Performance Indicators

**Fig. 1:**
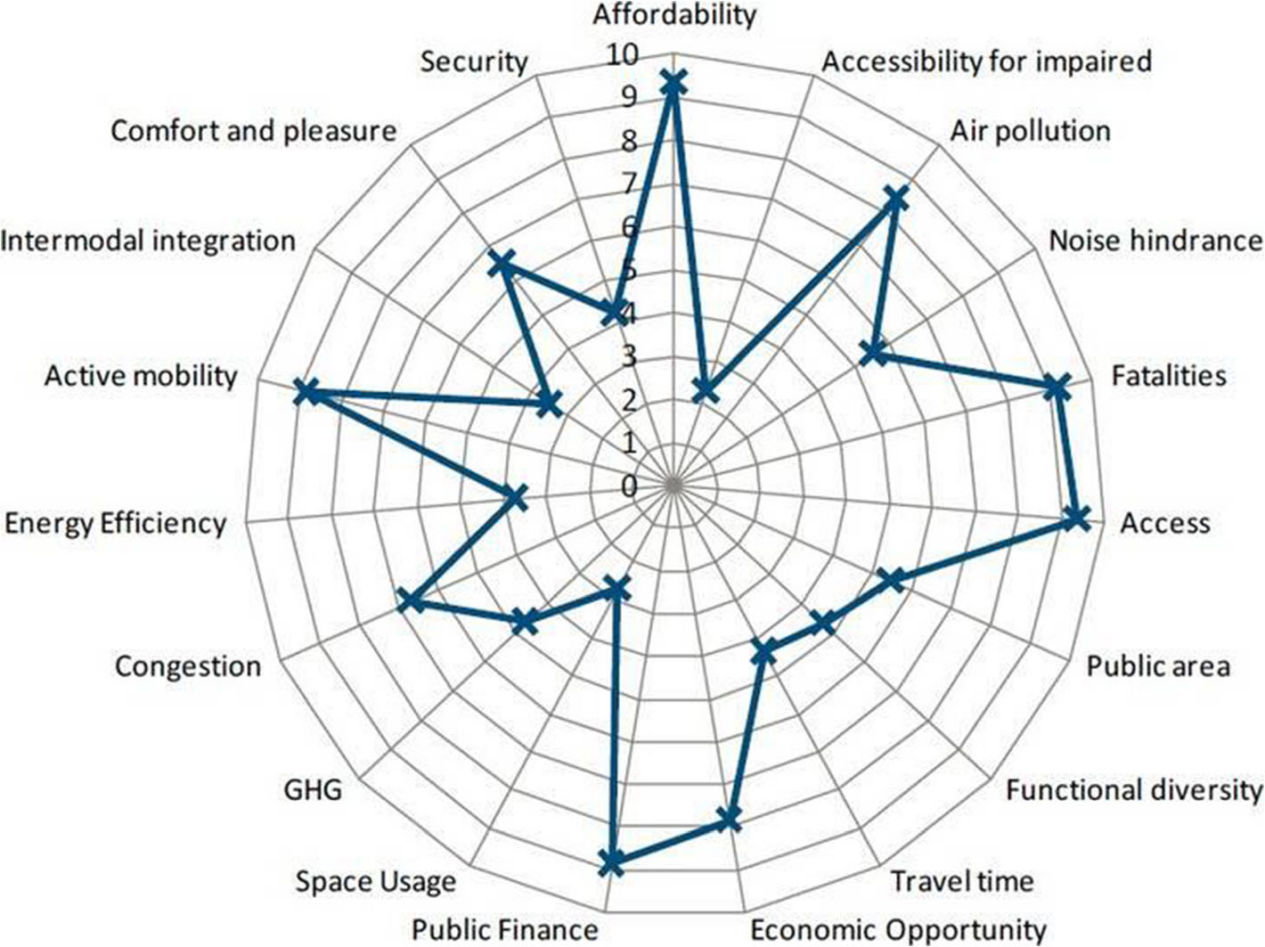
The 19 Sustainable Urban Mobility Indicators used by the World Business Council for Sustainable Development (WBCSD) for assessing sustainable mobility measures [16]

A good basis to help define KPIs for CASE mobility applications was the Sustainable Urban Mobility Indicators, or SUMI, which have been developed and applied in several European urban areas as part of their SUMP-related implementations [15, 17]. Also, the World Business Council for Sustainable Development has recently formulated and proposed 19 Sustainable Urban Mobility KPIs [16]. These KPIs are shown in Fig. 1 where the name of each KPI indicates also its content and nature. These KPIs have been tested in five urban areas (Bangkok, Campinas, Chengdu, Hamburg, and Lisbon). A later study [17] provided technical support to some 53 EU urban areas to use the WBCSD indicators and it developed a benchmarking functionality for these KPIs. Finally, another EU-funded project, the C-MOBILE (https://c-mobile-project.eu/) also developed a number of SUMIs.

There are, therefore, numerous suggestions and study results for a number of KPIs that are close or directly related to CASE mobility applications evaluation. The final selection and use of such indicators would be made by taking into account the local conditions and data availability.

As a result of the above considerations, for the case of Greece, and based on the experience from the applications in the projects mentioned in section 2.1 above, and the existing data sources in Greece [9], a number of sixteen CASE mobility evaluation KPIs have resulted, and these are shown in Table 1.

### Matrix evaluation

The result of the application of CMME analysis is given below as an example of the application of the matrix evaluation methodology. It concerns the evaluation of two alternative CASE mobility measures for the center of Thessaloniki, Greece. The two applications are aimed at reducing the traffic congestion in the CBD (central business district) by reducing the flows of cars that drive through it. One alternative was the introduction of a new intelligent traffic control system along the Egnatia Avenue (a main road artery that leads to the CBD) that would deter traffic flows from transiting through the center and the other was the delineation of a number of traffic-free zones, enforced through CASE mobility applications, in parts of the center. The “intelligent traffic control” strategy would be based on an advanced traffic control system that had been implemented along this artery a few years earlier [8]. The system (implemented under the scientific supervision of HIT/CERTH) consisted of a number of “intelligent” traffic control lights and traffic detectors operating under an intelligent control software supplied by two consulting companies SWARCOMIZ (Swarco-Mizar of Italy), and INFOTRIP (Greece).

The evaluation process involved the construction of two CMME tables— one for each alternative. Table 2 shows the CMME as completed (by a group of 4 experts), for the alternative of “traffic-free zones” in the CBD. A similar table was constructed for the second alternative tested, i.e., the “intelligent” traffic control strategy but is not shown here in the interests of space. As it can be seen in Table 2, the total score for the alternative tested there is 380. The corresponding score for the second alternative (i.e., the “intelligent” traffic control strategy) came to a total of 512. This indicated that the second alternative ranked higher overall scores and it was therefore judged preferable to the traffic-free zones one.

**Table 2:** Example of a “CMME evaluation” table for the assessment of a CASE mobility application involving traffic-free zones in the CBD of the city of Thessaloniki (a similar table was constructed for the alternative CASE mobility application involving intelligent traffic control)

	*Potential impact or evaluation criteria*	CASE mobility application elements (for both applications):												Row totals (sum of scores × weights)
		Intelligent—traffic control		Travel info		Dynamic route guidance		Road user charging		e-surveillance		Freight deliveries mgmt.		
*1*	Improved safety levels	6	2	3	2	7	2	2	2	1	2	3	2	44
*2*	Reduced CO_2_ emissions	3	3	2	3	7	3	6	3	2	3	5	3	75
*3*	Access to PT services	6	1	7	1	6	1	-	1	6	1	-	1	25
*4*	Reduced congestion	9	3	4	3	8	3	9	3	4	3	4	3	114
*5*	Smooth and seamless journeys	5	2	6	2	4	2	3	2	4	2	3	2	50
*6*	City liveability	4	3	1	3	2	3	8	3	4	3	5	3	72
	Column totals (left: sum of rows 1–6/right: sum of the products: scores × weights, rows 1–6)	33	76	23	46	34	79	28	79	21	46	20	54	380

Note: (a) The gray left-hand columns under each element contain the score given to the corresponding criterion (potential impact) on a scale 1–10. The right-hand columns show the weight of the corresponding impact (scale from 1 to 3). For each cell, the product of these two numbers (not shown in the Table) is summed to the bottom row and last right-hand column. (b) The elements of this particular CASE application that appear as the most “impactful” are “dynamic route guidance” and “road user charging” (weighted score 79), followed by “intelligent predictive traffic management” (weighted score 76). Finally, (c) the specific application is expected to have maximum impact on “reduced congestion” (weighted score 114) followed by “reduced CO_2_ emissions” (score 75) and so on

## Discussion

Co-operative, Autonomous, Shared, and Electric mobility systems in our future urban areas (referred to in this paper, as “CASE mobility”) will be advanced forms of urban mobility based on both new vehicle technologies—like the fully automatic (driverless) vehicles—as well as on Intelligent Transport Systems (ITS) applications based on Information Technology (IT) applications such as Artificial Intelligence (AI), the Internet of Things (IoT) or the big data collection infrastructures. CASE mobility will also include new models of operation with new mobility services (shared transport, etc.). Compared to the more traditional ITS applications that are being implemented in our urban areas for the last decade, CASE mobility applications will be more complicated, more horizontally integrated and based on multiple technological or service innovations. So, the evaluation and assessment of such new CASE mobility applications has to be adapted so as to be able to cover “wholesale” (horizontally) the assessment of the relevant impacts.

The key message of the work presented in this paper is that a basic (ad minimum) coordinated and uniformly applied approach is necessary for the assessment of CASE mobility measures. Such an approach is necessary to exist before the full-scale deployment of CASE mobility applications something that is expected to come during the new decade (starting after the return to “normality” from the COVID-19 pandemic).

An appropriate legal and statutory framework will also be necessary both for the collection of such data on a regular basis and for the implementation of specific evaluation/assessment frameworks such as the ones described here. For the first, the usual way is to legislate “urban CASE mobility observatories” along the lines of ELTIS, the European Urban Mobility Observatory (www.eltis.org). These “observatories” could be formed within existing organizations or be new ones. Besides the regular collection of data and mobility monitoring, they would also assist in the implementation of common evaluation and assessment methodologies for CASE mobility applications. On a wider scale, such frameworks at national level would greatly enhance the chances for an overall harmonized CASE mobility systems evaluation system at European-level that would secure fair, open, transparent, and comparable evaluations.

## Conclusions

This paper—based on the review of experience gained from the application of new ITS and CASE mobility technologies in Greece over the last decade—proposes the use of four “horizontal” types of evaluation namely Key Performance Indicators (KPIs), interviews, safety audits, and matrix evaluation. All four of these methodologies can be adapted and constructed in their final details according to the specific features and conditions of each application case also based on the variety of existing bibliographical references many of which are mentioned in this paper. We have shown here, as an example, the potential way of applying two of these methods based on experience and data from relevant applications in Greece.

The key feature is—as always—the collection of proper data. A regular system of relevant data collection would be a best-case option for any urban area that applies CASE-related applications. The minimum data required to be collected in such a system would be those necessary for the definition of the set of KPIs suggested in Table 1. Over and above those, such a regular system of data collection would need to contain traffic management related data (for use in the traffic control center algorithms and optimization models) as well as data for feeding into a dynamic traveler information system that most urban areas should now examine the possibility of installing. Various, and of advanced technology, IT applications exist for such data collection (e.g., through a network of sensors as well as through social-media derived data or “cooperating” floating cars).

## Data Availability

Data sharing not applicable to this article as no datasets were generated or analyzed during the current study.

## Abbreviations

AI: Artificial Intelligence
CAPITAL: Collaborative capacity Programme on ITS Training-education and Liaison
CASE: Co-operative, Autonomous, Shared, and Electric (mobility)
CBD: Central Business District
C-ITS: Connected Intelligent Transport Systems
CMME: CASE Mobility Matrix Evaluation
C-MOBILE: Accelerating C-ITS Mobility Innovation and depLoyment in Europe
CO_GISTICS: Cooperative logistics for sustainable mobility of goods
CO_2_: Carbon dioxide
CONDUITS: Coordination of network descriptors for Urban Intelligent Transportation Systems
ELTIS: European Urban Mobility Observatory
ENoLL: European Network of Living Labs
EU: European Union
GIS: Geographic Information System
HIT/CERTH: Hellenic Institute of Transport/Center for Research and Technology Hellas
IoT: Internet of Things
IT: Information Technology
ITS: Intelligent Transport Systems
KPI: Key Performance Indicators
MCA: Multi-criteria analysis
MICA: Motivational Interviewing Competency Assessment
NOx: Nitro oxides
PMx: Particulate Matter of x micrometers width
PT: Public transport
SHOW: SHared automation Operating models for Worldwide adoption (EU funded research project)
SOTIF: Safety of the Intended Functionality
SUMI: Sustainable Urban Mobility Indicators
SUMP: Sustainable Urban Mobility Plans
TREDIT: Transeuropean Consultants for Transport, Development and Information Technology
TSMLL: Thessaloniki Smart Mobility Living Lab
UMP: Urban Mobility Package
VRU: Vulnerable road users
WBCSD: World Business Council for Sustainable Development
